# Reducing stomatal density by expression of a synthetic epidermal patterning factor increases leaf intrinsic water use efficiency and reduces plant water use in a C_4_ crop

**DOI:** 10.1093/jxb/erae289

**Published:** 2024-07-18

**Authors:** John N Ferguson, Peter Schmuker, Anna Dmitrieva, Truyen Quach, Tieling Zhang, Zhengxiang Ge, Natalya Nersesian, Shirley J Sato, Tom E Clemente, Andrew D B Leakey

**Affiliations:** Institute for Genomic Biology, University of Illinois Urbana-Champaign, Urbana, IL 61801, USA; Institute for Genomic Biology, University of Illinois Urbana-Champaign, Urbana, IL 61801, USA; Institute for Genomic Biology, University of Illinois Urbana-Champaign, Urbana, IL 61801, USA; Department of Agronomy and Horticulture, University of Nebraska, Lincoln, NE, USA; Department of Agronomy and Horticulture, University of Nebraska, Lincoln, NE, USA; Department of Agronomy and Horticulture, University of Nebraska, Lincoln, NE, USA; Department of Agronomy and Horticulture, University of Nebraska, Lincoln, NE, USA; Department of Agronomy and Horticulture, University of Nebraska, Lincoln, NE, USA; Department of Agronomy and Horticulture, University of Nebraska, Lincoln, NE, USA; Institute for Genomic Biology, University of Illinois Urbana-Champaign, Urbana, IL 61801, USA; Department of Plant Biology, University of Illinois Urbana-Champaign, Urbana, IL 61801, USA; Department of Crop Sciences, University of Illinois at Urbana-Champaign, Urbana, IL 61801, USA; University of Western Australia, Australia

**Keywords:** C_4_ photosynthesis, *Sorghum bicolor*, stomata, water-use efficiency

## Abstract

Enhancing crop water use efficiency (WUE) is a key target trait for climatic resilience and expanding cultivation on marginal lands. Engineering lower stomatal density to reduce stomatal conductance (*g*_s_) has improved WUE in multiple C_3_ crop species. However, reducing *g*_s_ in C_3_ species often reduces photosynthetic carbon gain. A different response is expected in C_4_ plants because they possess specialized anatomy and biochemistry which concentrates CO_2_ at the site of fixation. This modifies the relationship of photosynthesis (*A*_N_) with intracellular CO_2_ concentration (*c*_i_), such that photosynthesis is CO_2_ saturated and reductions in *g*_s_ are unlikely to limit *A*_N_. To test this hypothesis, genetic strategies were investigated to reduce stomatal density in the C_4_ crop sorghum. Constitutive expression of a synthetic epidermal patterning factor (EPF) transgenic allele in sorghum led to reduced stomatal densities, reduced *g*_s_, reduced plant water use, and avoidance of stress during a period of water deprivation. In addition, moderate reduction in stomatal density did not increase stomatal limitation to *A*_N_. However, these positive outcomes were associated with negative pleiotropic effects on reproductive development and photosynthetic capacity. Avoiding pleiotropy by targeting expression of the transgene to specific tissues could provide a pathway to improved agronomic outcomes.

## Teaser

Stomatal density can be engineered to improve water use efficiency and avoid drought stress without impairing photosynthetic CO_2_ uptake in a model C_4_ crop.

## Introduction

Water availability is a critical factor limiting crop productivity worldwide (Boyer, 1982). Water use efficiency (WUE) has been recognized as an important trait for crop improvement for over a century (Briggs and Shantz, 1917) because it describes how much productivity can be achieved per unit of water used by the crop. WUE is key to terrestrial plant function because it reflects the inevitable trade-off between losing water vapour from leaves to the atmosphere, while stomata are open, to allow photosynthetic CO_2_ uptake. Climate change is impacting precipitation patterns and vapour pressure deficit, which in turn impact water availability and demand in cropping systems (Chiang *et al.*, 2021; Sadok *et al.*, 2021). *In silico* modelling that incorporates historical yields and projected environmental conditions suggests that drought events will continue to be a key driver of yield losses in the future (Webber *et al.*, 2018). Water supplies for irrigation are limited and unsustainable (WWAP, 2015). Demand is increasing for agricultural products from marginal lands (Gelfand *et al.*, 2013; Khanna *et al.*, 2021). Consequently, there is renewed focus on whether crop productivity, sustainability, and resilience can be enhanced through improvements to WUE (Bailey-Serres *et al.*, 2019; Hatfield and Dold, 2019; Leakey *et al.*, 2019; Sales *et al.*, 2021).

Intrinsic WUE (iWUE) is defined as the ratio of the rate of net photosynthetic CO_2_ assimilation (*A*_N_) relative to stomatal conductance (*g*_s_). Improving iWUE can be achieved by increasing *A*_N_ without a matching increase in *g*_s_, or by decreasing *g*_s_ without a matching decrease in *A*_N_. However, many studies of natural and engineered genetic variation within a broad diversity of plant species have shown *A*_N_ and *g*_s_ to be correlated (Leakey *et al.*, 2019; Deans *et al.*, 2020). Consistent with this expectation, in C_3_ species, theory and experimentation have shown that a decrease in stomatal density drives lower *g*_s_ and improves iWUE, but often at the cost of lower *A*_N_ (Wang *et al.*, 2016; Hughes *et al.*, 2017; Caine *et al.*, 2019; Dunn *et al.*, 2019; Mohammed *et al.*, 2019). So, de-coupling of *A*_N_ and *g*_s_ in transgenic plants is rare and, when it is achieved, iWUE is often reduced rather than increased (Flexas *et al.*, 2013).

Meanwhile, crop genotypes selected by breeders for greater iWUE have often been found to be innately less productive, as part of a generally conservative growth syndrome (Condon *et al.*, 2004). Although successes have resulted from screening stable carbon isotopes as a proxy to select for high iWUE in C_3_ species (Rebetzke *et al.*, 2006), scepticism remains about the potential for meaningful improvement of WUE without a concomitant hit on yield (Condon *et al.*, 2004; Blum, 2005; Sinclair, 2012).

Recent innovations have emerged that open the door for improving WUE and productivity in C_4_ crops through lower *g*_s_ via a reduction in stomatal density. These include novel high-throughput phenotyping tools to capture stomatal density and WUE traits that are accelerating the speed and scale of experimentation (Ferguson *et al.*, 2021; Pignon *et al*., 2021; Xie *et al.*, 2021). Importantly, unlike C_3_ crops, C_4_ feedstocks possess a carbon-concentrating mechanism that increases [CO_2_] in the bundle sheath cells where the primary photosynthetic enzyme Rubisco is located, resulting in concentrations significantly greater than atmospheric [CO_2_] (von Caemmerer and Furbank, 2003). Consequently, the relationship between *A*_N_ and intercellular [CO_2_] (*c*_i_) features a much steeper initial slope and a sharper inflection point than that observed in C_3_ species (Leakey *et al.*, 2009). Atmospheric [CO_2_] has risen from 370 ppm in 2000 to 417 ppm in 2023 (https://gml.noaa.gov/ccgg/trends/). As a result, the *c*_i_ at which photosynthesis operates in C_4_ crops is very close to saturation, which raises it above the *A*/*c*_i_ curve inflection point (Leakey *et al.*, 2006; Ghannoum, 2008; Markelz *et al.*, 2011). This means that more CO_2_ is entering the leaf than is required to maintain *A*_N_ and, theoretically, *g*_s_ could be reduced to increase iWUE with little (<2%) to no increase in the stomatal limitation to *A*_N_ (SL: Leakey *et al.*, 2019). The magnitude of reductions in stomatal density needed to achieve an optimal *g*_s_ is unknown, but too large a reduction in stomatal density would lower *c*_i_ to the point that it would fall below the inflection point on the *A*/*c*_i_ curve, where SL becomes substantial (Leakey *et al.*, 2019). Mechanistic crop modelling suggests that a genetic strategy that translates to a reduction in *g*_s_ by 20% would significantly increase yields of a C_4_ feedstock, such as sorghum, with the greatest gains occurring in marginal land locations where yields are currently low (Leakey *et al.*, 2019). Overexpression of STOMATAL DENSITY AND DISTRIBUTION 1 (SDD1) in maize provided evidence in support of this approach, driving reduced stomatal density and *g*_s_, without a decrease in *A*_N_ (Liu *et al.*, 2015). However, pleiotropy was observed, with the low stomatal density plants also having greater photosynthetic capacity than the wild type (WT). The consequences of overexpressing SDD1 for development, productivity, and WUE at the whole-plant scale were not reported.

Whole-plant transpiration rates regulate passive nitrogen (N) uptake from the soil (Niu *et al.*, 2007; Matsunami *et al.*, 2010; Kunrath *et al.*, 2020). Therefore, a potential drawback of a genetic strategy to improve WUE via a reduction in *g*_s_ is that N flux in the transpiration stream might be reduced, which in turn would negatively impact photosynthetic capacity and productivity. A significant fraction of leaf N is invested in photosynthetic proteins, with C_4_ species allocating less N to Rubisco than C_3_ species, but more N to other soluble proteins and thylakoid components (Ghannoum *et al.*, 2011). However, with ectopic expression of EPIDERMAL PATTERNING FACTOR 1 (EPF1) in the C_3_ species wheat, barley, and rice, photosynthetic capacity was reported not to decline (Hughes *et al.*, 2017; Caine *et al.*, 2019; Dunn *et al.*, 2019).

Testing the proposed approach to developing C_4_ crops with greater WUE is aided by the discovery of a network of genes that regulate leaf epidermal cell fate and, thereby, stomatal density in C_3_ species, because their C_4_ orthologues can be used as initial candidate genes for testing (McKown and Bergmann, 2020). Most studies that have manipulated stomatal density in C_3_ species have achieved this by overexpressing the native form of EPF1, a negative regulator of stomatal development (Harrison *et al.*, 2020), or down-regulating EPIDERMAL PATTERNING FACTOR-LIKE 9 (EPFL9 or stomagen), a positive regulator of stomatal development (Karavolias *et al.*, 2023). The EPF family of secreted signalling peptides function within the epidermal cell layer to regulate stomatal patterning (Hara *et al.*, 2007; Hunt and Gray, 2009). Various EPF fusions swapping the loop and scaffold regions of EPF2 and EPFL9 in Arabidopsis demonstrated that the loop region confers the functional specificity of EPFs (Ohki *et al.*, 2011). EPF peptides trigger a mitogen-activated protein (MAP) kinase signalling pathway that regulates the stability of SPEECHLESS (SPCH). SPCH is a basic helix–loop–helix (bHLH) transcription factor that contributes to the determination of cell division and fate transitions (Lau *et al.*, 2014). MAP kinases phosphorylate and destabilize SPCH, preventing the initiation of stomatal lineage cells; consequently, overexpressing EPF genes negatively regulates stomatal density via a decrease in SPCH protein levels (Kumari *et al.*, 2014). Recent evidence from C_3_ monocotyledonous species, such as barley, rice, and wheat, suggests that despite substantial differences in leaf expansion and stomatal development, native grass EPFs act in a similar way to the EPFs of the dicotyledonous model Arabidopsis, regulating entry to and progression through the stomatal cell lineage (Hughes *et al.*, 2017; Caine *et al.*, 2019; Dunn *et al.*, 2019; Mohammed *et al.*, 2019). On the other hand, the role of other regulators of stomatal and leaf development are not always strictly conserved across species, and few stomatal development genes have been tested in C_4_ species (Liu *et al.*, 2009; Raissig *et al.*, 2016; Schuler *et al.*, 2018; Hughes and Langdale, 2022).

Sorghum has a high WUE and is well adapted to xeric environments (Rooney, 2014). It is an important model C_4_ species, with significant contributions to the bioeconomy as a feedstock for food, feed, and industrial applications (Paterson *et al.*, 2009; Morris *et al*., 2013; Castro *et al.*, 2015). Various models have shown that in high-yielding environments, a reduced water use trait can protect yield during soil moisture deficits (Sinclair *et al.*, 2005; Truong *et al.*, 2017).

This study tested two genetic designs to reduce stomatal density in sorghum, to address the knowledge gap about how engineering reduced stomatal density would impact photosynthetic physiology and whole-plant function in a C_4_ species. In the first design, the native sorghum EPF1 (SbEPF1) was constitutively expressed under control of the sugarcane ubiquitin4 (Ubi4) promoter (Wei *et al.*, 2003). In the second design, a fusion element was synthesized that combined elements of the sorghum orthologues of AtEPF2 and AtEPFL9 (SbEPF_syn_) and was placed under control of the Ubi4 promoter. Comparing sorghum events with reduced stomatal density versus WT controls addressed whether reduced stomatal density: (i) can drive reductions in *g*_s_ without significantly increasing SL; (ii) drives pleiotropic effects on leaf physiology or plant development; (iii) produces leaf-level reductions in *g*_s_ that scale to reduced whole-plant water use; and (iv) alters whole-plant biomass production.

## Materials and methods

### Assembly of binary vectors

The leucine-rich repeat, receptor-like kinase ERECTA corresponding interacting partner EPF1 is a negative regulator of stomatal development in Arabidopsis (Hara *et al.*, 2007). An ectopic expression cassette was designed to misexpress EPF1 in sorghum, and was designated pPTN1337. The ORF of the sorghum homologue of AtEPF1 (NM_127657), Sobic006G233600.1/SbiTx43006G248600.1, was synthesized (GeneScript, USA), which incorporated the gene model’s 5'- and 3'-untranslated region (UTR) elements. The synthesized ORF with its corresponding UTRs was subcloned between the sugarcane Ubi4 promoter (Wei *et al.*, 2003) and the 3' UTR of cauliflower mosaic virus 35S transcript (T35S). The derived expression cassette was subsequently cloned into the binary vector of pPZP212 (Hajdukiewicz *et al.*, 1994) and the resultant vector was designated pPTN1337 (Supplementary Fig. S1A).

A second vector was designed for the expression of a fusion peptide that comprises a domain from STOMAGEN (EPFL9; Hunt *et al.*, 2010; Kondo *et al.*, 2010) and EPF2 (Hara *et al.*, 2009). This fusion consists of amino acid residues 26–219 from the sorghum homologue of AtEPF2 (NM_103147), Sobic006G104400.1/SbiTx43006G109800.1, that resides downstream of residues 24–37 of the sorghum homologue of STOMAGEN (AtNP_193033.1), Sobic003G299800.1/SbiTx43003G4311400.1. The fusion element is imbedded within the 5' and 3' UTRs of the sorghum EPF2 gene model (Supplementary Fig. S2). This element was synthesized (GenScript, USA) and subsequently subcloned between the sugarcane Ubi4 promoter and T35S terminator. The expression cassette was then cloned into pPZP212 (Hajdukiewicz *et al.*, 1994) and the derived binary vector was designated pPTN1338 (Supplementary Fig. S3A).

The derived binary vectors were introduced into *Agrobacterium tumefaciens* strain NTL4/pTiKPSF_2_ (Luo *et al.*, 2001), and the resultant transconjugants were used to transform the grain sorghum genotype Tx430 as previously described (Howe *et al.*, 2006; Guo *et al.*, 2015).

### Initial genotyping and phenotyping of transgenic events

**Fig. 1.**
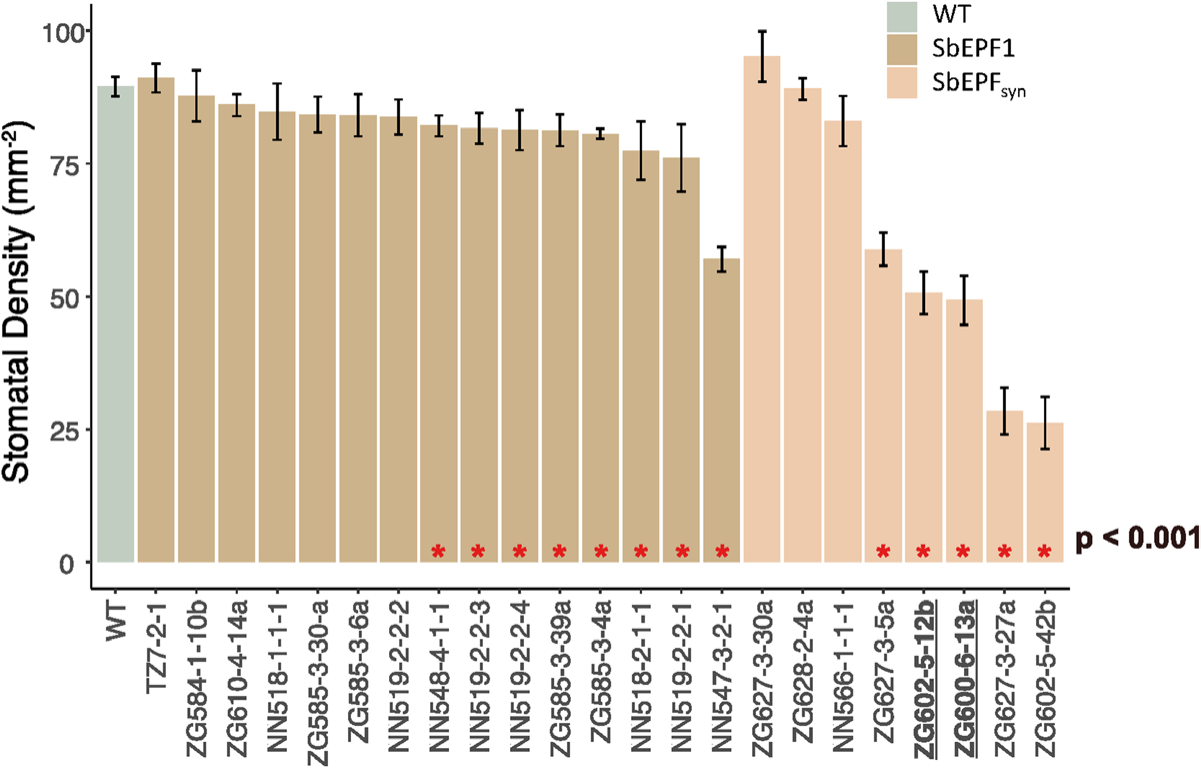
Phenotypic screen of transgenic plants. Abaxial stomatal density of independent transgenic events of sorghum (A) ubiquitously expressing the sorghum orthologue of EPIDERMAL PATTERNING FACTOR-1 (SbEPF1) and (B) ubiquitously expressing a fusion element synthesized from elements of the sorghum orthologues of EPIDERMAL PATTERNING FACTOR-2 (EPF2) and EPIDERMAL PATTERNING FACTOR-LIKE 9 (EPFL9) (SbEPF_syn_). The events that displayed significantly different stomatal densities compared with the wild type (WT), according to a post-hoc Tukey test following a one-way ANOVA, are denoted by red asterisks. The independent events of SbEPF_syn_ that were carried forward for further investigation are highlighted in bold and underlined (ZG602-5-12b and ZG600-6-13a).

Progeny derived from selfed lineages of the obtained transgenic events were (i) assessed for the presence of the plant selectable marker allele via an neomycin phosphotransferase II (NPTII) ELISA according to the manufacturer’s instructions (Agdia Inc., Elkhart, IN, USA) and (ii) phenotyped for changes in stomatal density by optical tomography (Xie *et al.*, 2021; see below for details). Phenotyping was performed on 15 independent, positive events of SbEPF1 and eight independent, positive transgenic events of SbEPF_syn_. Of these, two independent events carrying the SbEPF_syn_ allele with significantly reduced stomatal density were selected for further characterization (Fig. 1A).

The subset of the transgenic events, including the two SbEPF_syn_ events, were characterized by both Southern blot and RNA gel blot analyses as previously described (Howe *et al.*, 2006; Mall *et al.*, 2011). Here, total genomic DNA was digested with *Eco*RI for Southern blot analysis (pPTN1338 events), and the membranes for both northern and Southern hybridizations were probed with a ^32^P-labelled 719 bp element that carried a region of the fusion ORF of pPTN1338 (Supplementary Fig. S3B, C). For the RNA gel blot analysis conducted on a set of pPTN1337 events (Supplementary Fig. S1B), the membrane was hybridized with an ~570 bp region of the SbEPF1 ORF spanning into the 3' UTR.

### Experimental design for detailed evaluation of SbEPF_syn_

Plants were planted, grown, and phenotyped within the greenhouse facility at the University of Illinois at Urbana-Champaign (latitude 40.11°, longitude –88.21°). The greenhouse conditions were set to a 16 h photoperiod (07.00–23.00 h) with supplementary light provided by high pressure sodium and metal halide growth lamps. The target day/night temperature was set to 28/21 °C.

WT (Tx430) and T_2_ transgenic lineages were sown directly into trays of 4 cm deep cells filled with Sunshine™ organic germinating mix (SunGro, Agawam, MA, USA). At the three-leaf stage, the presence of the transgene was verified in the manner described previously. Additionally, T-DNA copy number analysis of NPTII was performed relative to a known single-copy sorghum gene amplicon by iDNA genetics (Norwich, UK), which confirmed the homozygosity of the transgenic allele in the respective progeny lineages going forward.

Ten replicate plants from both the WT and the two independent events of SbEPF_syn_ (ZG602-5-12b and ZG602-6-13a) were transplanted into 17.5 litre pots containing a known mass of Sunshine Mix #4 professional growing mix (SunGro, Agawam, MA, USA). The mass of each pot and of the soil within each pot was recorded to allow later calculation of the volumetric relative soil water content (% rSWC), as previously described (Ferguson *et al.*, 2018). Apart from 9 d mid-growing cycle when water was withheld to perform a ‘dry-down’ experiment (details below), plants were kept well watered and supplemented with liquid Nature’s Source 3-1-1 NPK fertilizer (Ball DPF LLC, Sherman, TX, USA) bi-weekly. To avoid potential spatial bias, the positions of the pots were randomized within blocks of the greenhouse space every 3 d across the full duration of the study, as well as every day during the ‘dry-down’ portion of the study.

Phenotypic sample and data collection occurred in three phases. First, at the sixth leaf stage, the three most recently fully expanded leaves (i.e. leaves four, five, and six) were assessed for stomatal density. At that time, light-saturated rates of leaf photosynthetic gas exchange, photosynthetic capacity, N content, and specific leaf area (SLA) were also measured on the youngest, fully expanded leaf. Second, at the ninth leaf stage, a ‘dry-down’ experiment was performed by withholding water for 9 d from the plants of each genotype. Two days prior to withholding water, whole-plant leaf area was assessed. During the ‘dry-down’ experiment, whole-plant water use and photosynthetic leaf gas exchange were measured daily. Lastly, at plant maturity, above-ground biomass production was determined.

### Leaf gas exchange measurements

When leaf six had just fully expanded, the response of net photosynthetic CO_2_ assimilation (*A*_N_) to the concentration of leaf intercellular CO_2_ (*c*_i_) was measured using a LI-COR 6800 infrared gas exchange system equipped with a standard 6 cm^2^ cuvette (LI-COR Inc., Lincoln, NE, USA). Data collection occurred between 08.00 h and 15.00, just prior to harvesting tissue from the same leaves for the other leaf physiological traits. Environmental conditions were set at: 27 °C, 65% relative humidity, 1800 μmol m^–2^ s^–1^ photosynthetic photon flux density (PPFD), 400 µmol mol^–1^ CO_2_ concentration, and 400 µmol s^–1^ flow rate. After full photosynthetic induction was established, *A*_N_, *g*_s_, and *c*_*i*_ were recorded as the leaf was exposed to a series of stepwise changes in sample CO_2_ concentrations of: 400, 200, 50, 150, 300, 400, 500, 600, 700, 800, and 1200 µmol mol^–1^. A custom R function was used for modelling *A*_N_–*c*_i_ response curves following (von Caemmerer, 2000) to estimate the maximum rate of carboxylation by phosphoenolpyruvate carboxylase (PEPC; *V*_pmax_) and the asymptote of the *A*_N_–*c*_i_ curve (*V*_max_), as described previously (Markelz *et al.*, 2011).

During each day of the water withdrawal experiment, *A*_N_ and *g*_s_ were measured between 08.30 h and 13.00 h on the youngest fully expanded leaf. Measurements were performed using LI-6400 gas exchange systems (LI-COR Inc.) equipped with a 2 cm×3 cm LED cuvette, and conditions in the gas exchange cuvette were as described for the *A*_N_–*c*_i_ response measurements.

### Leaf stomatal density, specific leaf area, and nitrogen content

A Nanofocus µsurf explorer optical topometer (Nanofocus, Oberhausen, Germany) was used to assess stomatal patterning, as described previously (Haus *et al.*, 2015; Ferguson *et al.*, 2021). When leaf six had just fully expanded, four fields of view (800 × 800 µm each in size) arranged in a transect between the mid-rib and margin at a position midway along the length of leaves four, five, and six were scanned at ×20 magnification on both the abaxial and adaxial surfaces. 3D reconstructions were converted to 2D grey-scale images for analysis. Stomatal density was determined using the cell counter feature in ImageJ (Abràmoff *et al.*, 2006). In addition, four samples per genotype from leaf six were re-scanned at ×50 magnification on the abaxial surface. Six stomatal complexes were then randomly selected to measure their widths and lengths from the most extreme points in ImageJ (Abràmoff *et al.*, 2006). These trait values were then averaged for each sample.

At the same time, leaf discs were sampled from the sixth leaf for estimation of SLA and tissue N content, as described previously (Markelz *et al.*, 2011).

### Whole-plant water use

To estimate water consumption during the ‘dry-down’ experiment, plants were soaked to ~100% rSWC and subsequently not watered for 9 d. Each pot was weighed daily during this period. These data were used to calculate rSWC whilst accounting for pot weight at the start of the experiment, and plant mass measured on three replicates harvested on the day that water withholding started.

### Above-ground biomass production

Two days prior to the water withdrawal period, whole-plant leaf area was determined as the sum of the width multiplied by the length of every leaf. This non-destructive estimation of leaf area is highly correlated to conventional measurements of leaf area in maize (Pearce *et al.*, 1975).

At full maturity, plants from both watering treatments were harvested just above the soil level and dried at 60 °C for 2 weeks before being weighed.

### Statistical analyses

To test for overall phenotypic differences from the WT in both the initial screening of all transgenic events and the subsequent detailed evaluation of events ZG602-5-12b and ZG602-6-13a for SbEPF2_syn_ under well-watered conditions, a one-way ANOVA comparison of means test was performed. To then determine which events were significantly different from the the WT, a post-hoc Tukey test was performed.

To test for differences in genotype effects on stomatal density between leaf positions (i.e. leaf four, five, or six), and to test for differences in genotype effects on above-ground biomass between watering treatments (i.e. fully watered at all times versus plants that experienced the ‘dry-down’ treatment), two-way fully factorial ANOVA tests were performed. To test for differences between the two transgenic lines, a specific contrast test was used for a given leaf surface across the three leaf positions.

To determine genotypic differences in the response of rSWC, *g*_s_, and *A*_N_ to declining water availability, two-way fully factorial ANOVA tests were performed where time was treated as a repeated measure. Post-hoc Tukey tests were subsequently performed to ascertain on which days and between which genotypes significant differences occurred.

All ANOVA tests were performed using the base lm() function in R. Where multiple subsamples were measured within a replicate plant (i.e. stomatal density from multiple fields of view per leaf), an average was calculated for the replicate plant and this was the input for all statistical tests. Least-squares means and SEs for all groups from each test were computed using the lsmeans() function from the lsmeans R package (Lenth, 2016). The least-square means and SEs are reported in the associated bar and line plots. Post-hoc Tukey tests were performed using the HSD.test() function from the agricolae R package (de Mendiburu and Simon, 2015, Preprint). All figures were produced using the ggplot2 R package (Wickham, 2009) with post-processing in Affinity Designer (Serif, Nottingham, UK).

## Results

### Peptide alignments of Arabidopsis and sorghum epidermal patterning factors SbEPF1, SbEPF2, and SbEPF9

Global alignments of the sorghum gene models that encode the epidermal patterning factors, EPF1, EPF2, and EPF9, with their corresponding Arabidopsis homologues reveal varying degrees of homology at the protein level. The sorghum and Arabidopsis EPFL9 peptides share ~47% identity with 66% similarity (Supplementary Fig. S4), with the highest degree of identity at the C-terminal regionand core motif of the protein. An alignment of the sorghum and Arabidopsis EPF1 peptides displays ~25% identity, with 31% similarity, with the highest degree of relationship also at the C-terminal region of the peptide (Supplementary Fig. S5). The respective EPF2 proteins show 28% identity and 41% similarity, and, like the other epidermal patterning factors, the shared alignments occur at the C-terminal region of the peptide (Supplementary Fig. S6).

### Phenotypic and molecular screens of SbEPF1 and SbEPF_syn_

Eight of the 15 independent events of SbEPF1 had significantly reduced abaxial stomatal density compared with the WT. However, in all except one case (NN547-3-2-1, –26% relative to the WT) the reductions in stomatal density were very modest (–8% to –15%; Fig. 1). By contrast, five of the eight EPF_syn_ events had significantly lower stomatal density than the WT, and all five had much greater reductions in stomatal density (–34% to –71%; Fig. 1). Two independent events (ZG602-5-12b and ZG600-6-13a), each carrying a single homozygous copy of the transgene and with similar reductions in stomatal density compared with the WT (–43% and –45%), were selected for further investigation at the T_2_ stage. These lines are referred to as ‘12b’ and ‘13a’.

### Stomatal patterning and light-saturated leaf gas exchange of EPF_syn_

**Fig. 2.**
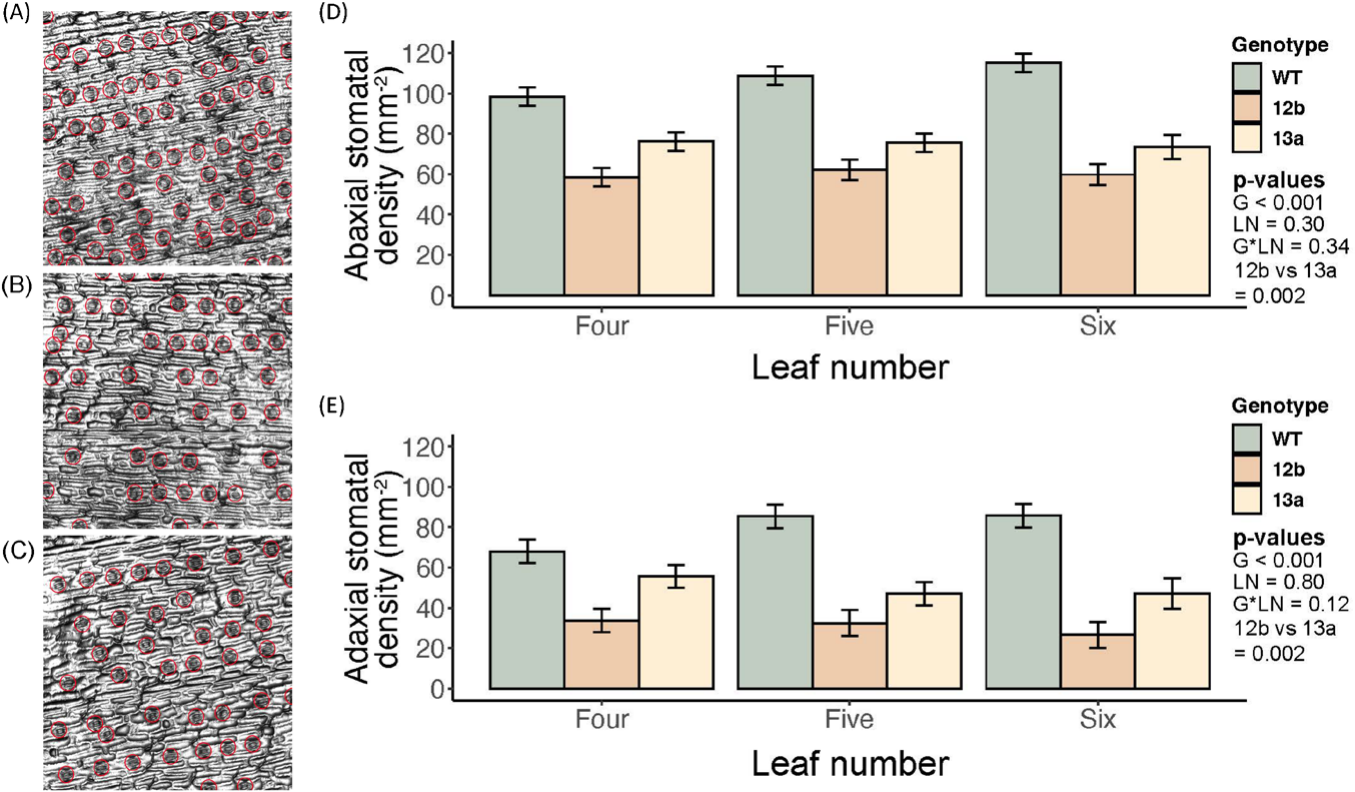
Stomatal patterning phenotype of engineered plants. Representative micrographs of the abaxial leaf surface of: (A) the wild type (WT), (B) SbEPF_syn_ line ZG602-5-12b (12b), and (C) SbEPF_syn_ line ZG600-6-13a (13a), along with the associated (D) abaxial stomatal density and (E) adaxial stomatal density at three leaf positions on the main culm (three, four, and five) for each genotype. Bars represent least square means of stomatal density for each grouping, where the errors bar represent the associated SEs. For the abaxial and the adaxial surface, the *P*-values from each term in a two-way ANOVA with an interaction term are inset.

In both lines of EPF_syn_, stomatal density was confirmed to be significantly lower than in the WT on both the abaxial (*P*<0.001) and adaxial leaf surfaces (*P*<0.001) of fully expanded leaves at positions four, five, and six on the main culm (Fig. 2). On average across the leaf positions, stomatal density of line 12b was significantly lower than that of line 13a for both the adaxial (*P*=0.002) and abaxial leaf surface (*P*=0.002). The average reduction in stomatal density was greater on the adaxial surface (–61% in line 12b, –36% in line 13a) than on the abaxial surface (–43% in line 12b, –30% in line 13a), and this effect was consistent across leaf positions. All genotypes had significantly greater stomatal density on the abaxial versus adaxial leaf surface. Stomatal complex size was significantly greater in line 12b (*P*<0.05) and trended towards being larger in line 13a (Supplementary Table S1).

**Fig. 3.**
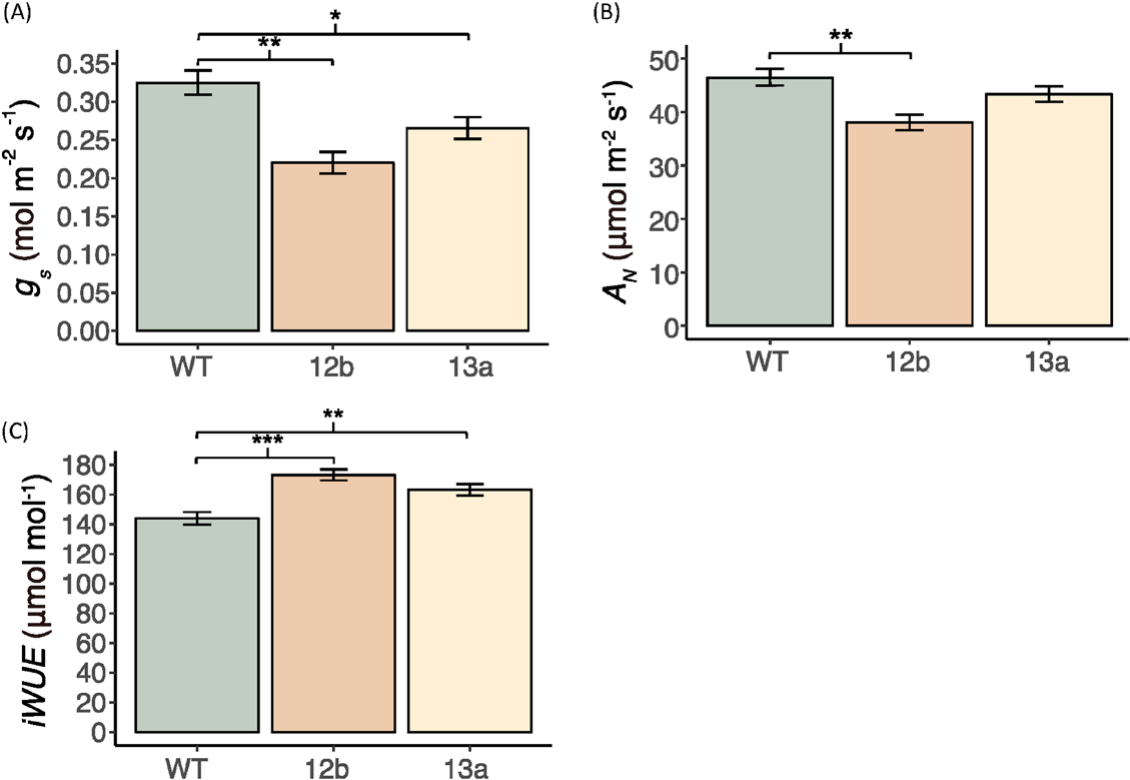
Leaf gas exchange of engineered plants. Light-saturated (A) stomatal conductance (*g*_s_), (B) net photosynthetic assimilation of CO_2_ (*A*_N_), and (C) intrinsic water-use efficiency (iWUE) of the sixth leaf on the culm when it was the youngest fully expanded leaf of the wild type (WT), SbEPF_syn_ line ZG602-5-12b (12b), and SbEPF_syn_ line ZG600-6-13a (13a). Bars represent least square means, and error bars represent associated SEs. Significant differences between genotypes are denoted as *P>0.05, ** *P*>0.01, or *** *P*>0.001.

Steady-state, light-saturated *g*_s_ was significantly lower in EPF_syn_ compared with the WT, with line 12b showing a stronger effect (–32%, *P*<0.01) than line 13a (–18%, *P*<0.05; Fig. 3A). In line 12b, the stronger reduction in *g*_s_ was accompanied by a reduction in *A*_N_ compared with the WT (–22%, *P*<0.01; Fig. 3B). For both lines of EPF_syn_, the reduction in *g*_s_ led the ratio of *A*_N_ to *g*_s_ (i.e. iWUE) to be significantly greater than for the WT (20% for line 12b, 13% for line 13a; *P*<0.01; Fig. 3C).

**Fig. 4.**
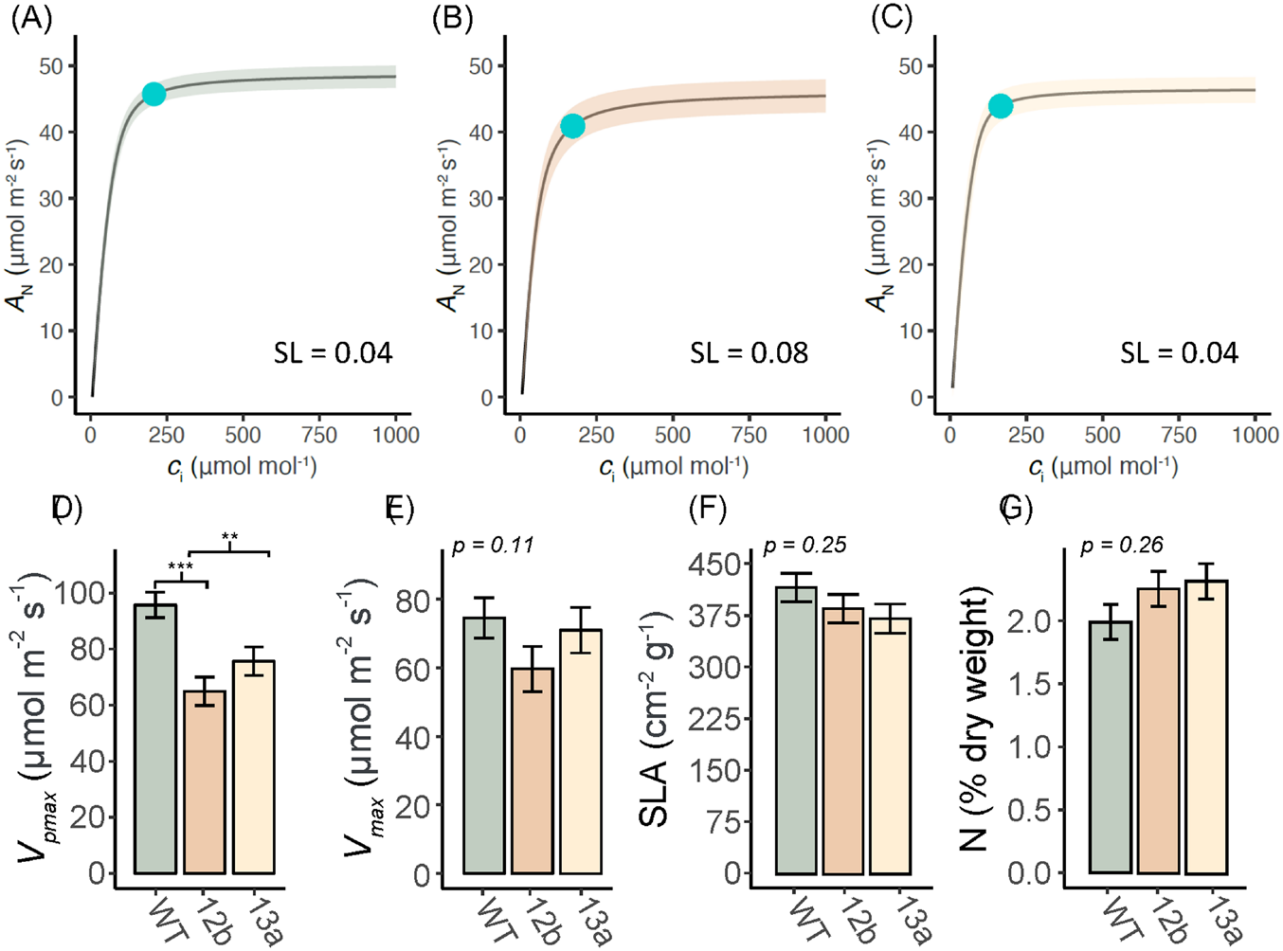
Photosynthetic capacity of engineered plants. Fitted average photosynthetic/CO_2_ response (*A/c*_i_) curves of (A) the wild type (WT), (B) SbEPF_syn_ line ZG602-5-12b (12b), and (C) SbEPF_syn_ line ZG600-6-13a (13a), along with (D) the apparent maximum rate of carboxylation by PEPC (*V*_pmax_), (A) the asymptote of the *A/c*_i_ curve (*V*_max_), (F) specific leaf area (SLA), and (G) leaf nitrogen (N) content of the sixth leaf on the culm when it was the youngest fully expanded leaf. For *A/c*_i_ curves, the mean fit is represented by the solid line and the SE is denoted by the shaded area. The stomatal limitation to *A*_N_ at ambient [CO_2_] (SL) is reported for each treatment. The operating point of photosynthesis at ambient [CO_2_] is shown as a blue dot on the *A/c*_i_ curve. Bars represent least square means, and error bars represent associated SEs. The *P*-values from associated one-way ANOVA tests are inset. Where a significant effect was detected, the differences between the transgenic lines and the WT according to post-hoc testing is shown.

The observed changes in light-saturated gas exchange of EPF_syn_ versus the WT were the result of changes in both stomatal and mesophyll limitations to photosynthesis. As expected, SL was low (0.04; Fig. 4A) in the WT, and was unchanged in line 13a (0.04; Fig. 4C). This corresponded to the operating point of photosynthesis (blue dot) remaining above the inflection point on the *A*/*c*_i_ curve when *g*_s_ was more moderately reduced in line 13a compared with the WT. In line 12b, with its stronger reduction in stomatal density, SL was greater (0.08; Fig. 4B) than in the WT and the operating point of photosynthesis was at the inflection point on the *A*/*c*_i_ curve. EPF_syn_ plants of both lines had lower photosynthetic capacity than the WT as a result of significantly reduced apparent capacity for carboxylation by PEPC (*V*_pmax_; –32% in line 12b, –21% in line 13a, *P*<0.01; Fig. 4D) and also a marginally significant reduction in the combined apparent capacity for carboxylation by Rubisco and PEP regeneration by pyruvate orthophosphate dikinase (PPDK; *V*_max_) in line 12b (–20%, *P*=0.11; Fig. 4E). There were no significant differences between EPF_syn_ lines and the WT in SLA (*P*=0.25; Fig. 4F) or leaf N concentration (*P*=0.26; Fig. 4F).

### Interactions between leaf gas exchange, whole-plant water use, and drought stress

**Fig. 5.**
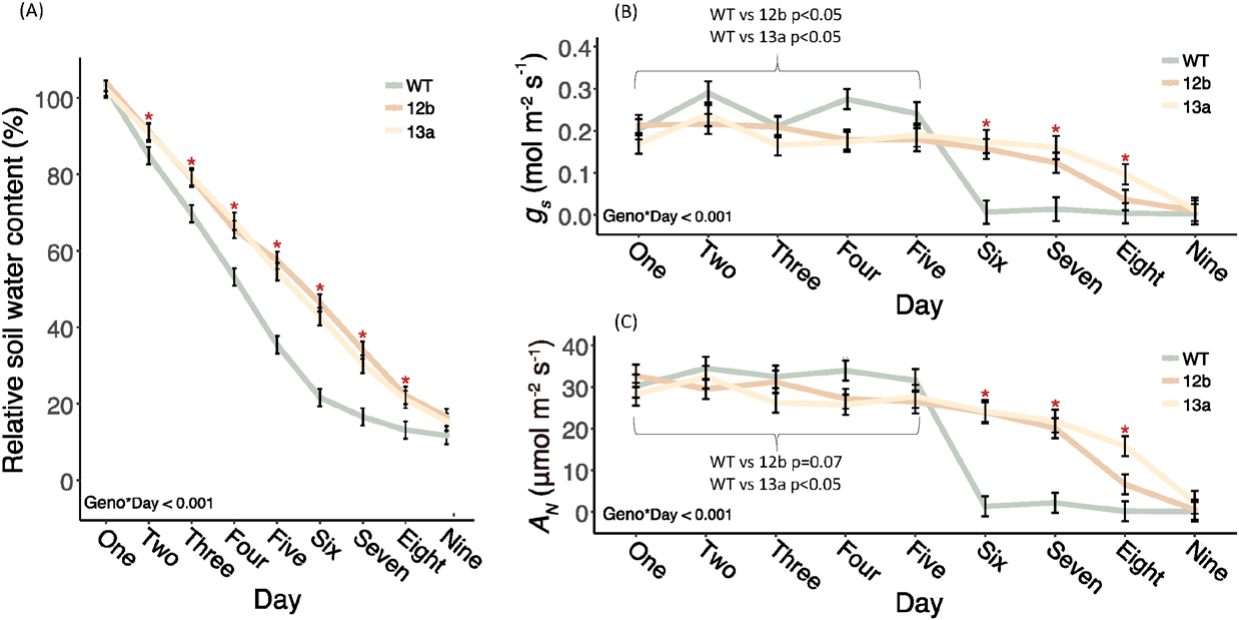
Water use and leaf gas exchange of engineered plants during a dry-down period. The response of (A) percentage relative soil water content, (B) stomatal conductance (*g*_s_), and (C) net photosynthetic assimilation of CO_2_ (*A*_N_) to a 9 d water withdrawal period for wild-type (WT), SbEPF_syn_ line ZG602-5-12b (12b), and SbEPF_syn_ line ZG600-6-13a (13a) sorghum. Points and errors bars represent least square means and SEs, respectively. *P*-values associated with repeated measure ANOVAs are inset. Days where transgenic events ZG602-5-12b (12b) and ZG600-6-13a (13a) were significantly different from the WT for all traits are indicated by red asterisks. The statistical results of pairwise tests between each transgenic line and the WT for a specific contrast of average *g*_s_ and *A*_N_ on the first 5 d of the experiment is shown by the inset bracket.

The rSWC was significantly greater in pots of both EPF_syn_ lines compared with the WT as early as day 2 of the dry-down experiment (Fig. 5A). This water saving increased in magnitude until day 6, at which point WT plants had almost exhausted the available soil water and their rate of soil drying slowed dramatically. Since initial rates of soil drying were 30–34% lower in EPF_syn_ lines compared with the WT, they were sustained for longer before water supply became limiting, only displaying a slight reduction in the rate of water use on day 9, at which point pot soil moisture was almost exhausted for EPF_syn_ lines and the WT (Fig. 5A). Considering the potential influence of plant size on rates of water use, a trend towards lower total leaf area (7–12%) in the EPF_syn_ lines compared with the WT was observed at the beginning of the dry-down experiment, but the effect was not statistically significant (*P*=0.60; Supplementary Fig. S7).

**Fig. 6.**
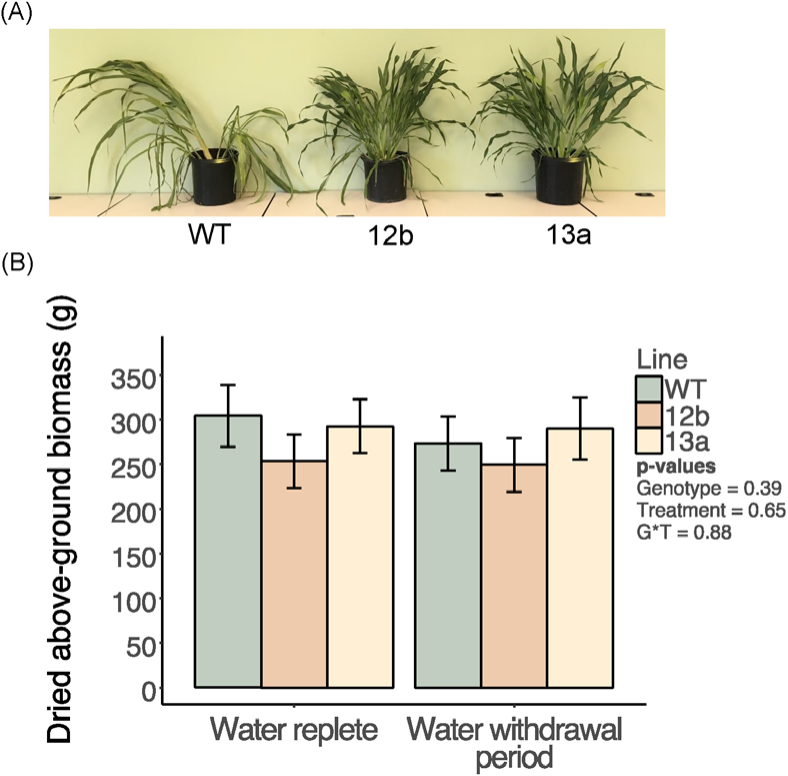
Whole-plant phenotypic characters of engineered plants. (A) Photographs of representative plants of wild-type (WT), SbEPF_syn_ line ZG602-5-12b (12b), and SbEPF_syn_ line ZG600-6-13a (13a) plants on day 8 of the water withdrawal period. (B) Total dried above-ground biomass of all genotypes grown under water-replete conditions or subjected to the water withdrawal period. Bars represent least square means, and error bars represent associated SEs. *P*-values associated with each term from an associated two-way ANOVA are inset.

During the first 5 d of the dry-down experiment, when high rates of water use were sustained in all pots, *g*_s_ was 17% and 24% lower in the EPF_syn_ lines compared with the WT (*P*<0.05; Fig. 5B). This was accompanied by more modest average reductions in *A*_N_ in line 12b (–10%, *P*=0.07) and line 13a (–14%, *P*<0.05) (Fig. 5C). On day 6, a large and rapid decline in *A*_N_ and *g*_s_ occurred in the WT, while leaf gas exchange declined much more slowly in the EPF_syn_ lines (Fig. 5B, C). As a result, *g*_s_ and *A*_N_ were substantially greater for the EPF_syn_ lines compared with the WT from day 6 through day 8 (Fig. 5B, C). The difference in drought stress experienced by the plants was visually apparent, with the WT being significantly wilted at the end of the ‘dry-down’ experiment while the EPF_syn_ lines retained full turgor (Fig. 6A).

**Fig. 7.**
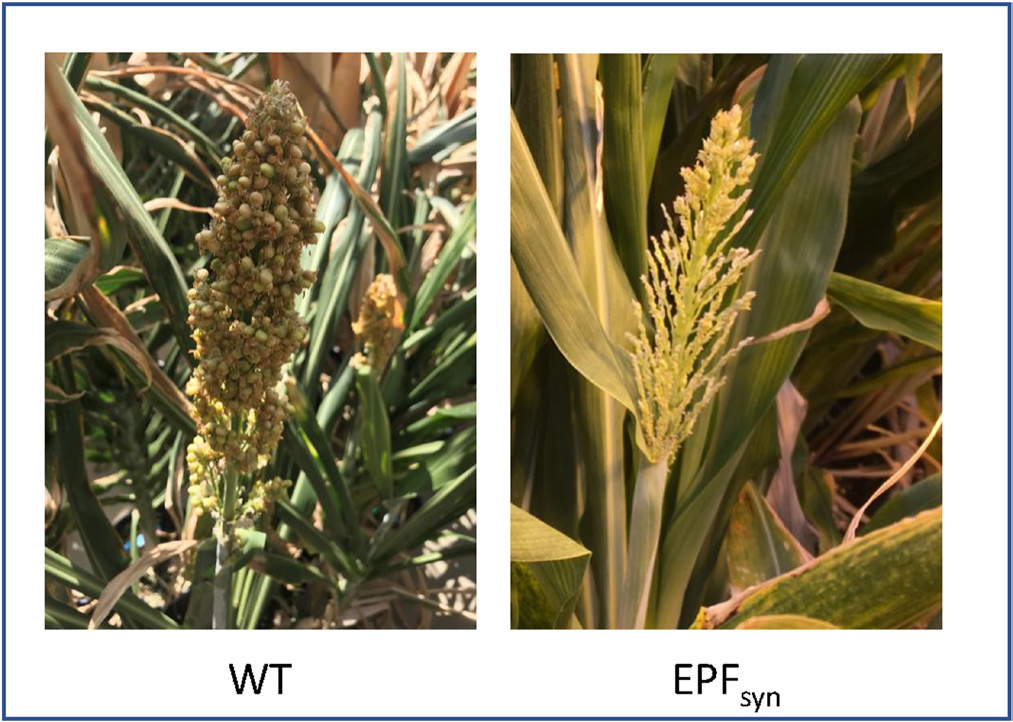
Photographs of representative panicles from wild-type sorghum (WT) and sorghum ubiquitously expressing a fusion element synthesized from elements of the sorghum orthologues of EPIDERMAL PATTERNING FACTOR-2 (EPF2) and EPIDERMAL PATTERNING FACTOR-LIKE 9 (EPFL9) (SbEPF_syn_).

The short period of drought stress relative to the overall growing period did not significantly alter biomass production (*P*=0.65; Fig. 6B). Across both watering regimes, both EPF_syn_ lines had slightly shorter internode lengths and overall heights (Fig. 6A); there was no significant difference among the genotypes in total dry, above-ground biomass at maturity (*P*=0.35; Fig. 6B). A pleiotropic response of impaired panicle and flower development was consistently observed in the EPF_syn_ lines, resulting in significantly reduced seed production (Fig. 7). Occasionally, some leaves of EPF_syn_ lines, but not of the WT, would exhibit a chlorotic strip that was 2–4 cm in width and contained almost no fully developed stomata (Supplementary Fig. S8).

## Discussion

This study contributes to our understanding of how engineered reductions in stomatal density impact leaf and whole-plant WUE in a model C_4_ species, sorghum, an important feedstock for the bioeconomy (Silva *et al.*, 2022). Ectopic expression of EPF1 has been shown to significantly reduce stomatal density and increase iWUE in a number of C_3_ species (Hughes *et al.*, 2017; Dunn *et al.*, 2019; Mohammed *et al.*, 2019). In sorghum, however, ectopic expression of EPF1 resulted in slight reductions in stomatal density (<15%) in all but one transformation event (Fig. 1). By contrast, ubiquitous expression of EPF_syn_ resulted in reductions of 34–71% in stomatal density relative to the WT (Fig. 1). Hence, deeper phenotypic characterizations were performed on events carrying the SbEPF2_syn_ transgenic allele to address the experimental objectives. Detailed evaluations of leaf gas exchange in two SbEPF2_syn_ events revealed improvements in iWUE compared with the WT, while highlighting the importance of achieving an intermediate reduction in stomatal density to lower *g*_s_ while still maintaining low SL (Figs 3, 4). Engineered gains in iWUE drove reductions in whole-plant water use without causing a loss in above-ground biomass production (Figs 5, 6). In addition, the study revealed the potential for pleiotropic effects on a number of developmental processes when engineering changes in stomatal development by ubiquitous expression of this SbEPF2_syn_ allele (Figs 4, 7). These observations provide a framework for future research to genetically enhance C_4_ plants in a manner that achieves the improvement in iWUE without an increase in SL, while avoiding unwanted pleiotropic effects on developmental processes other than stomatal patterning. Achieving this goal is a high priority because water limitation is so important to global crop productivity, especially in regions where agricultural production is vulnerable to climate variability, and because improving WUE is such a valuable, if challenging, target for crop improvement (Condon *et al.*, 2004; Blum, 2005; Leakey *et al.*, 2019).

### Balancing reductions in *g*_s_ with maintenance of stomatal limitation to *A*_N_

Analysis of *A*/*c*_i_ curves can be used to quantify SL, which describes how much lower the observed *A*_N_ is compared with a theoretical *A*_N_ where there is no resistance for CO_2_ entry into the leaf; that is, *c*_i_ equals the atmospheric [CO_2_] (Farquhar and Sharkey, 1982; Long and Bernacchi, 2003). The magnitude of changes in stomatal patterning and function in SbEPF_syn_ event 13a achieved the desired balance of reducing water loss through stomata without restricting CO_2_ entry to the leaf in a manner that limited photosynthesis. When comparing the light-saturated leaf gas exchange of line 13a with that of the WT, reduced stomatal density (–45%) was associated with lower *g*_s_ (–18%) and greater iWUE (+13%; Figs 2, 3). This outcome resulted: (i) from WT sorghum having low SL (0.04; Fig. 4A), as expected for a C_4_ crop under contemporary [CO_2_] of >410 ppm (Leakey *et al.*, 2019); and (ii) SL remained unchanged in line 13a (0.04). In other words, *A*_N_ was not reduced when lower stomatal density and *g*_s_ limited diffusion of CO_2_ into the leaf because the photosynthetic operating point remained above the inflection point of the *A*/*c*_i_ curve (Figs 3, 4C). By contrast, SbEPF2syn event 12b had a slightly stronger phenotype, where a greater reduction in stomatal density (–69%) and *g*_s_ (–32%) resulted in greater stomatal limitation to *A*_N_ (0.08) than in the WT (0.04; Figs 3, 4B). This resulted from the operating point of photosynthesis being at a lower *c*_i_, on the inflection point of the *A*/*c*_i_ curve, and contributed to *A*_N_ of event 12b being lower (–22%) than in the WT (Fig. 4B). These results validate the prediction, based on a leaf gas exchange model, that reducing *g*_s_ to an intermediate degree can enhance iWUE with very little to no penalty to carbon gain (Leakey *et al.*, 2019). It is important to emphasize that the steep inflection point of the C_4_*A*/*c*_i_ curve means that improving iWUE without a negative trade-off on photosynthetic carbon gain in sorghum, maize, sugarcane, miscanthus, and other C_4_ grasses will depend on precisely tuning the reduction in stomatal density to achieve greater iWUE without *g*_s_ and A_N_. Under the growth conditions used here, the optimal reduction in stomatal density lay somewhere between the stomatal phenotypes observed in SbEPF2_syn_ events 12b and 13a. As future atmospheric [CO_2_] continues to rise, progressively greater reductions in stomatal density will be needed to maintain the photosynthetic operating point at the optimal location just above the asymptote of the *A*/*c*_i_ curve.

The results of this study suggest further effort should be applied to enhance WUE in C_4_ crops by reducing stomatal conductance. While this study provides initial proof-of-concept for achieving that goal by reducing stomatal density, approaches to reduce stomatal complex size and/or the dynamic control of stomatal aperture by guard cells are also exciting possibilities for which the foundation is being laid (Des Marais *et al.*, 2014; Nunes *et al.*, 2023). There are also interactions between stomatal density and stomatal size that generally influence attempts to engineer greater iWUE, but are generally poorly understood (Lunn *et al.*, 2024; Supplementary Table S1). Being able to avoid an increase in SL while increasing iWUE in a C_4_ species is a result distinct from what is expected and often observed in C_3_ species (Leakey *et al.*, 2019). In C_3_ crops, the lack of a photosynthetic carbon-concentrating mechanism means that the *A*/*c*_i_ curve is less steep, and photosynthesis is not expected to be CO_2_ saturated today or later this century (Leakey *et al.*, 2006). Consequently, a modest loss in photosynthetic carbon gain under well-watered conditions results from engineering lower *g*_s_ to achieve enhanced iWUE (e.g. Yoo *et al.*, 2010; Franks *et al.*, 2015; Hughes *et al.*, 2017; Caine *et al.*, 2019). However, crop modelling suggests that this is a cost that is likely to be worthwhile for C_3_ crops growing in regions that are consistently water limited (Leakey *et al.*, 2019). Meanwhile, the possibility of avoiding the negative trade-off between water savings and carbon gain may allow low *g*_s_/high WUE C_4_ crops to enhance productivity across a wide range of growing conditions from consistently water-limited to only occasionally water-limited environments (Leakey *et al.*, 2019). However, as described below, significant challenges remain to be addressed to meet that goal.

### Pleiotropic effects on photosynthetic capacity from ubiquitous expression of SbEPF_syn_

In addition to the effects of *g*_s_, SL can be influenced by changes in photosynthetic capacity that alter the shape of the *A*/*c*_i_ curve (Markelz *et al.*, 2011). Both SbEPF_syn_ events had lower apparent photosynthetic capacity than the WT (Fig. 4). This produced a shallower *A*/*c*_i_ curve, shifted the inflection point to greater *c*_i_, and increased SL (0.08) relative to the WT (0.04). Together these responses explain the lower light-saturated *A*_N_ in event 12b compared with the WT (Fig. 4). In event 13a, the pleiotropic effects of SbEPF_syn_ on photosynthetic capacity were more moderate than in event 12b and had no consequences for *A*_N_.

Photosynthetic capacity, including the activity of PEPC, is strongly correlated to leaf N content in sorghum (Khan *et al.*, 2020). Moreover, in sorghum, maize, and rice, transpiration facilitates passive N flux, with consequences for leaf N content (Niu *et al.*, 2007; Matsunami *et al.*, 2010; Kunrath *et al.*, 2020). Therefore, it has been hypothesized that reducing transpiration to improve WUE might reduce N uptake and leaf N content (Hepworth *et al.*, 2015). However, leaf N content per unit leaf area was not significantly different between SbEPF_syn_ and the WT (Fig. 4G), indicating that some other mechanism must underlie the observed variation in *V*_pmax_. Changes in photosynthetic capacity have been observed in some other studies where expression of native stomatal patterning genes have been modified (Franks *et al.*, 2015; Liu *et al.*, 2015; Caine *et al.*, 2019; Dunn *et al.*, 2019). Additionally, wheat events ectopically expressing EPF1 had lower leaf porosity alongside reductions in stomatal density and stomatal conductance (Lundgren *et al.*, 2019). Further experimentation will be needed to determine the mechanisms underpinning changes in biochemical and anatomical limitation to photosynthetic capacity when engineering altered stomatal patterning.

### Pleiotropic effects of ubiquitously expressing SbEPF_syn_ on plant development

A significant off-target impact of ubiquitously expressing SbEPF_syn_ was impaired reproductive development (Fig. 7). EPF2 peptides are known to bind and regulate ERECTA to govern the division of protodermal cells into either pavement cells or stomatal complexes (Zoulias *et al.*, 2018). ERECTA-family receptor kinases coordinate stem cell functions between the epidermal and internal layers of the shoot apical meristem, and they are demonstrated to regulate floral patterning, fertility, and organ identity in addition to stomatal patterning in Arabidopsis (Shpak *et al.*, 2003; Cai *et al.*, 2017; Kimura *et al.*, 2018). So, it is possible that the ubiquitous expression of SbEPF_syn_ may have perturbed equivalent pathways in sorghum, leading to impaired panicle and flower development (Fig. 7). If so, enhancing iWUE in sorghum while avoiding pleiotropic effects on photosynthetic capacity and seed production might be achieved by the use of tissue-specific promoters that can limit expression of EPF_syn_ to the epidermis during early phases of leaf development.

### Whole-plant biomass production, water use, and drought avoidance

The overall biomass production of SbEPF_syn_ plants was equivalent to that of the WT (Fig. 6). As described above, this appears to have been due to the pleiotropic effects of expressing SbEPF_syn_ being focused on developmental processes, and under conditions of ample water supply the anatomical consequences had mild (event 12b) to no effect (event13a) on photosynthetic carbon gain.

Total plant water use is a function of both the rate of water use per unit leaf area and the total leaf area of the plant. The rate of water use by the two SbEPF_syn_ events was –30% and –34% lower than in the WT (Fig. 5A). Most of the water savings can be attributed to the reduction in *g*_s_, which averaged –20% and –23% lower in the two SbEPF_syn_ events compared with the WT, when averaged across all the dates of measurement on which water supply was not limiting (Fig. 5B). However, it seems likely that the –5% and –10% changes in total plant leaf area of the SbEPF_syn_ events compared with the WT also contributed to lower rates of water use, even if they were not resolved as statistically significant (Supplementary Fig. S7).

When water supply was withheld in the dry-down experiment, differences in rates of water use meant that SbEPF_syn_ plants took 9 d to exhaust the water supply in their pots versus 6 d for the WT (Fig. 5A). When the water supply ran out for WT plants, it triggered a substantial and rapid drop in *g*_s_ and *A*_N_ (Fig. 5B, C). It is noteworthy that *g*_s_ and *A*_N_ declined gradually between day 6 and day 9 in the SbEPF_syn_ events, compared with very abruptly decreasing between day 5 and day 6 in the WT (Fig. 5B, C). This is consistent with the interpretation of the *A*/*c*_i_ curve data described above, namely under mild, initial drought stress the photosynthetic operating point of SbEPF_syn_ plants required less of a drop in *g*_s_ and *c*_i_ to sit at or below the inflection point of the *A*/*c*_i_ curve, leading to a modest loss of carbon gain. Nevertheless, these experimental results support the notion that a low stomatal density, low *g*_s_, high iWUE strategy in a C_4_ crop results in greater carbon gain over the dry-down period as a whole, which may enhance photosynthetic carbon gain and biomass production in locations where water supply limits productivity (Leakey *et al.*, 2019). It is important to note that drought stress generally develops more rapidly and severely in pot experiments than under field conditions. For example, dry-down of the large soil volume at field locations in the Central USA can take weeks rather than a few days to develop (Leakey *et al*., 2004; Markelz *et al.*, 2011; sorghum drought). In addition, the dynamics of dry-down and re-wetting cause plant drought stress events to vary with soil type and climatic conditions. So, field trials and crop modelling will be needed to quantify the optimal phenotype of weak versus strong reductions in stomatal density and *g*_s_ across a range of growing conditions.

### Conservation of EPF function across C_3_ and C_4_ lineages

Overexpression of the native EPF1 genes in barley, rice, and wheat achieves significant reductions in stomatal densities that are like those or greater than those achieved via AtEPF1 overexpression in Arabidopsis—52% average reduction in barley across two events, 45% average reduction in rice across three events, and 70% average reduction in wheat across two events (Hughes *et al.*, 2017; Caine *et al.*, 2019; Dunn *et al.*, 2019). By comparison, the minor impact of ubiquitously expressing the native EPF1 on stomatal density in sorghum suggests that functional divergence or redundancy has occurred in sorghum and possibly other C_4_ grasses. The hallmark Kranz anatomy in C_4_ grasses is distinct from the leaf structure of C_3_ grasses, highlighting the possibility for the evolution of functional redundancy and/or novel function acquisition across these lineages. Phylogenetic divergence in stomatal characteristics between C_3_ and C_4_ grasses has been observed to mirror the evolution of the C_4_ photosynthetic pathway and local adaption (Taylor *et al.*, 2012; Lundgren *et al.*, 2014). Our results regarding SbEPF1 highlight the need to elucidate the genetic networks that underpin stomatal development in C_4_ grasses to understand how they differ between C_3_ dicots and grasses.

### Conclusion

In summary, this study provides support for prediction from modelling studies that engineering to reduce stomatal conductance can improve iWUE and act to lower plant water use without reducing biomass accumulation of a model C_4_ crop. However, it also highlights: (i) the potential for pleiotropic effects on a range of developmental processes when a mobile signalling peptide is expressed ubiquitously; and (ii) the currently limited understanding of the genetic basis for stomatal development in C_4_ grasses. These knowledge gaps will need to be addressed and more sophisticated engineering strategies and/or additional genes targeted to retain the desirable stomatal phenotypes observed here without the negative consequences of pleiotropic effects on the agronomics of the crop.

## Supplementary data

The following supplementary data are available at *JXB* online.

Fig. S1. Design of the binary vector and molecular characterization of transgenic plants expressing SbEPF1.

Fig. S2. Diagrammatic representation of the translational product of the fusion peptide expression cassette of SbEPFL9/SbEPF2 present in the binary vector pPTN1138.

Fig. S3. Design of the binary vector and molecular characterization of transgenic plants expressing synthetic EPF.

Fig. S4. The global alignment of SbEPFL9and AtEPFL9.

Fig. S5. The global alignment of SbEPF1and AtEPF1.

Fig. S6. The global alignment of SbEPF2and AtEPF2.

Fig. S7. Total leaf surface area of the wild type (WT), ZG602-5-12b, and ZG600-6-13a.

Fig. S8. Photographs of representative plants featuring the occasionally observed phenotype of a short white band on a mature leaf.

Table S1. Mean stomatal complex width and length of the wild type, ZG602-5-12b, and ZG600-6-13a.

erae289_suppl_Supplementary_Table_S1_Figures_S1-S8

## Data Availability

Primary data for this manuscript are available at: https://doi.org/10.13012/B2IDB-4017279_V1.
