# Supplementary material for: Reducing stomatal density by expression of a synthetic epidermal patterning factor increases leaf intrinsic water use efficiency and reduces plant water use in a C4 crop
Source: J Exp Bot. 2024 Jul 18;75(21):6823–36. doi: 10.1093/jxb/erae289 (PMC11565208; doi:10.1093/jxb/erae289)

Supplementary Table S1. Mean stomatal complex width and length of wildtype, ZG602-5-12b, and ZG600-6-13a. Standard errors of the mean are included in parenthesis. Asterisks denote a significant difference (*P* < 0.05) in trait value versus wildtype according to t-test. N = 4.

**Genotype**	**Stomatal complex width (μm)**	**Stomatal complex length (μm)**
WT	49.02 (2.89)	104.32 (2.09)
12b	67.01 (4.63) *	121.46 (9.45)
13a	52.53 (4.90)	113.53 (5.86)

Supp. Fig. S1

A

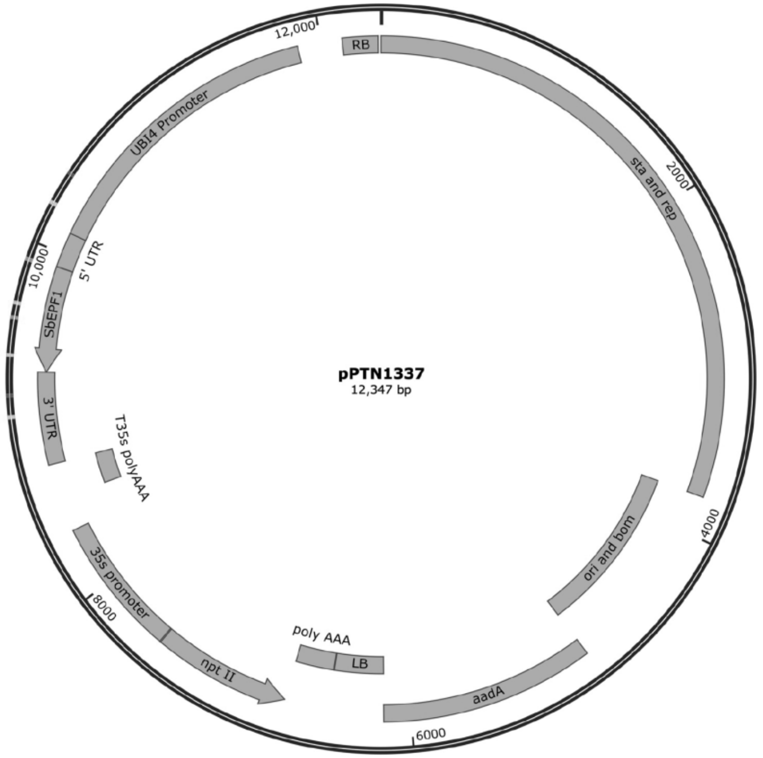

B

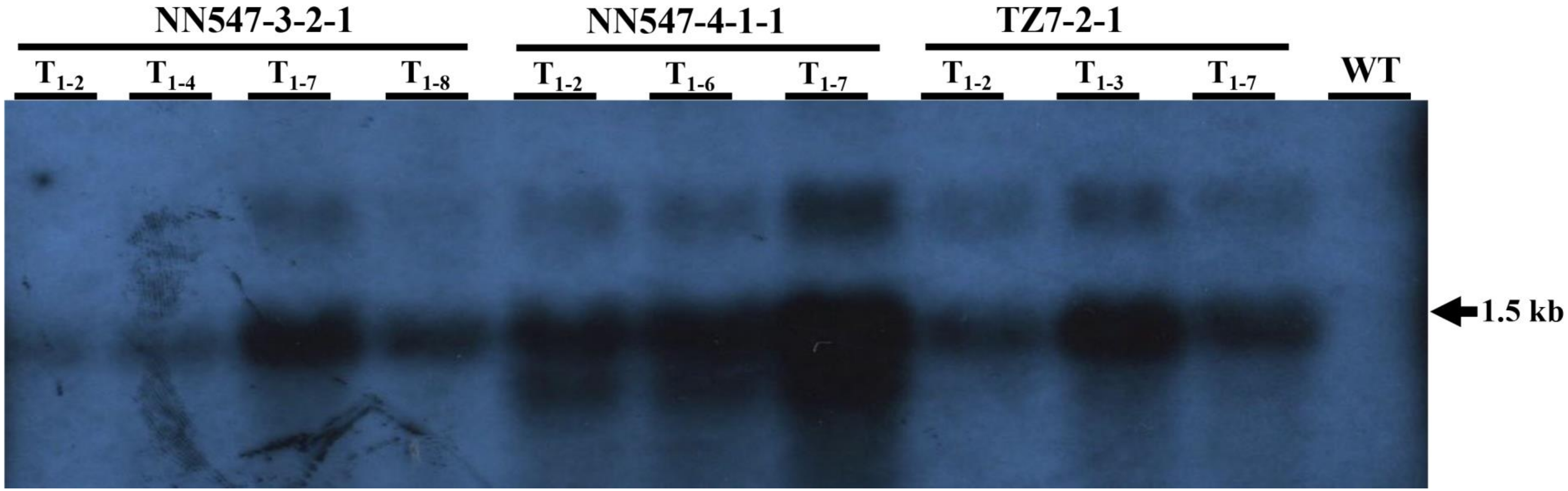

Supp. Fig. S2

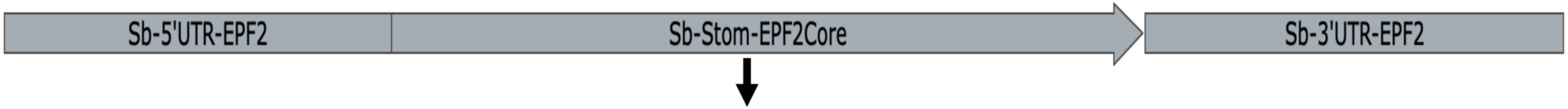

MGHALCSQGHNGRTSVVLVLLLLITTSSSTHVADGGVRTTPRAYGPGDAGVLDDAEAPAAAGGARRASSRATYKFPAD
RTSEDEGSLVVVEEDRRWWLKDRAATGSRLPDCAHACGPCSPCRRVIVSFRCALMASESCPIAYRCMCRGRFFRVPSP*

Supp. Fig. S3

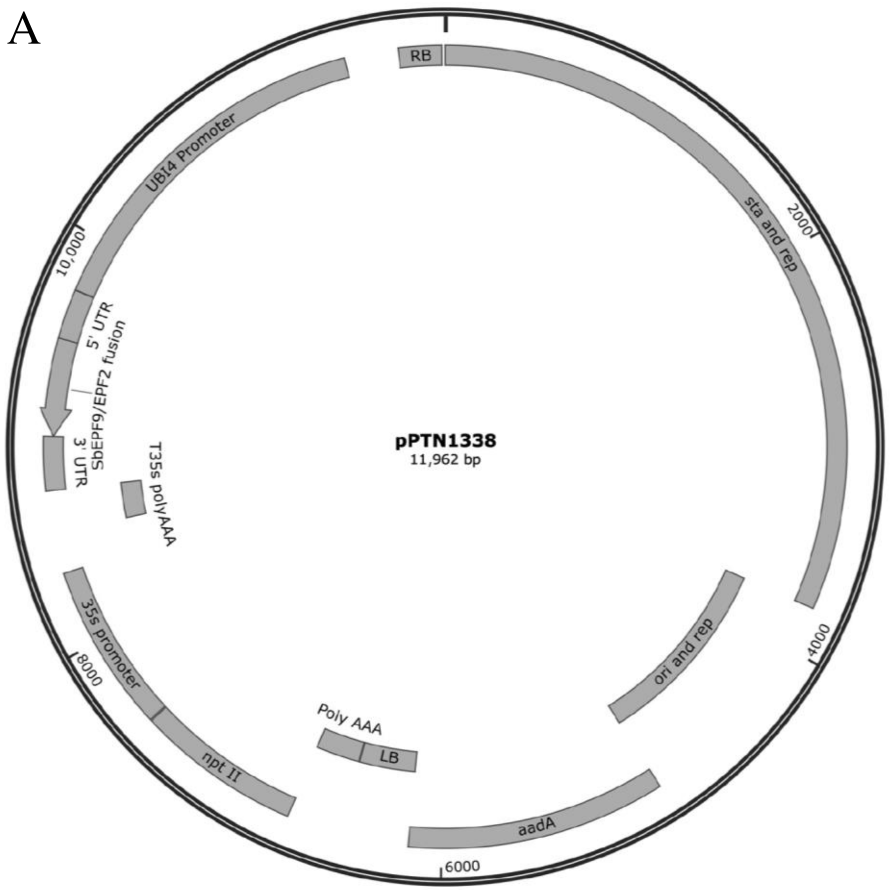
A
RB
sta and rep
2000
4000
ori and rep
aadA
6000
Poly AAA
LB
npt II
35s promoter
8000
T35s polyAAA
3' UTR
SbEPF9/EPF2 fusion
5' UTR
10,000
UBI4 Promoter
pPTN1338
11,962 bp

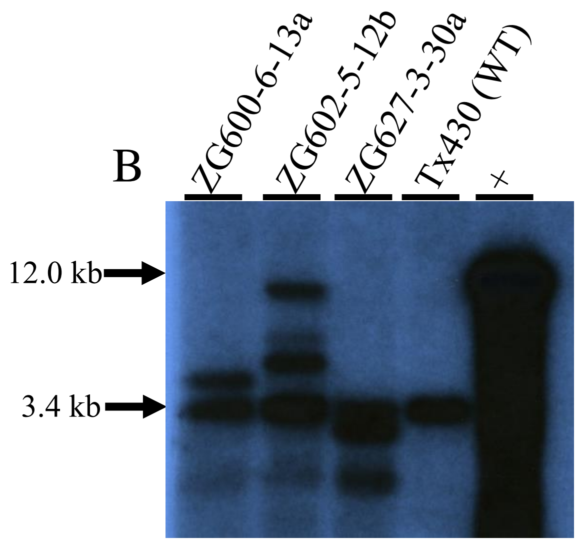
B
ZG600-6-13a
ZG602-5-12b
ZG627-3-30a
Tx430 (WT)
+
12.0 kb
3.4 kb

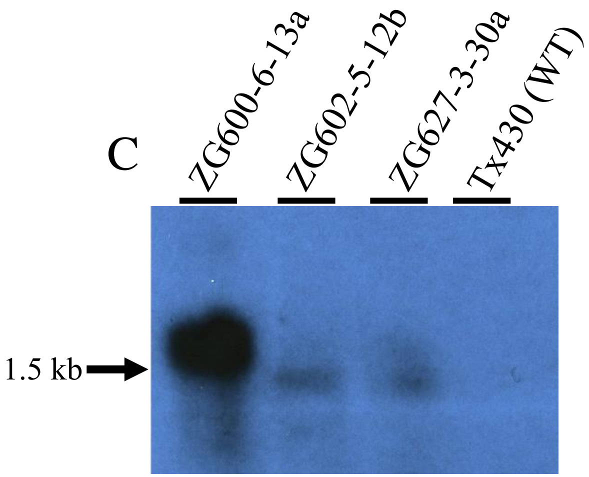
C
ZG600-6-13a
ZG602-5-12b
ZG627-3-30a
Tx430 (WT)
1.5 kb

Supp. Fig. S4

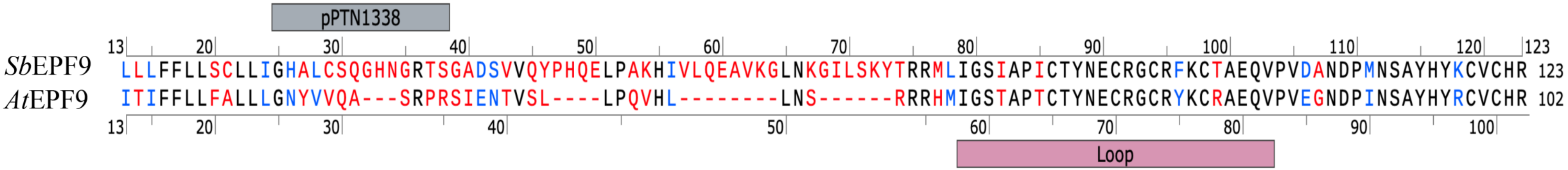

Supp. Fig. S5

```
SbEPF1 1    MLAIAAAIYMCTLGAETTYVQVQLARPRLAQHWSNRALRCLPGSRSQCSAAATPRRRLSS 60
AtEPF1 1    MKSLLLLAFF------------------LSFFFGSLLARHLPTSSH------------PS 30

SbEPF1 61   QHCEAYAVSLGLMGRHAGVVLLALTVVLLLAAVGDGIRPAPTAGASEMVHGSTTTTTEMV 120
AtEPF1 31   HH-------------HVGM----------------------TGAL--------------- 40

SbEPF1 121  VVAAPSAAQVQGKRSRGGKDDDLVLREEVVRATGSSLPDCSHACGACSPCSRVMVSFKC- 179
AtEPF1 40   ------------KRQR--------RRPDTVQVAGSRLPDCSHACGSCSPCRLVMVSFVCA 80

SbEPF1 180  SASEPLPCPMVYRCMCRGKCYPVPSS 205
AtEPF1 81   SVEEAETCPMAYKCMCNNKSYPVP-- 104
```

Supp. Fig. S6

```
SbEPF2 1     MR--MRESSACRCRPRIWWWSPAPAAAVVL 28
AtEPF2 1     MTKFVRKYMFC------------------- 11

SbEPF2 29    VLLLLITTSSSTHVADGGVRTTPRAYGPGD 58
AtEPF2 11    --LVLVFAACSLVV--NSIRTPP------- 30

SbEPF2 59    AGVLDDAEAPAAAGGARRASSRATYKFPAD 88
AtEPF2 30    ------LKNTVNGGEKKNADIEQAQTHHKK 54

SbEPF2 89    RTSEDEGSLVVVEEDRRWWLKDRAATGSRL 118
AtEPF2 55    EISKNGG----VE-------MEMYPTGSSL 73

SbEPF2 119   PDCAHACGPCSPCRRVIVSFRCALMASESC 148
AtEPF2 74    PDCSYACGACSPCKRVMISFECSV--AESC 101

SbEPF2 149   PIAYRCMCRGRFFRVPS-P 166
AtEPF2 102   SVIYRCTCRGRYYHVPSRA 120
```

Supp. Fig. S7

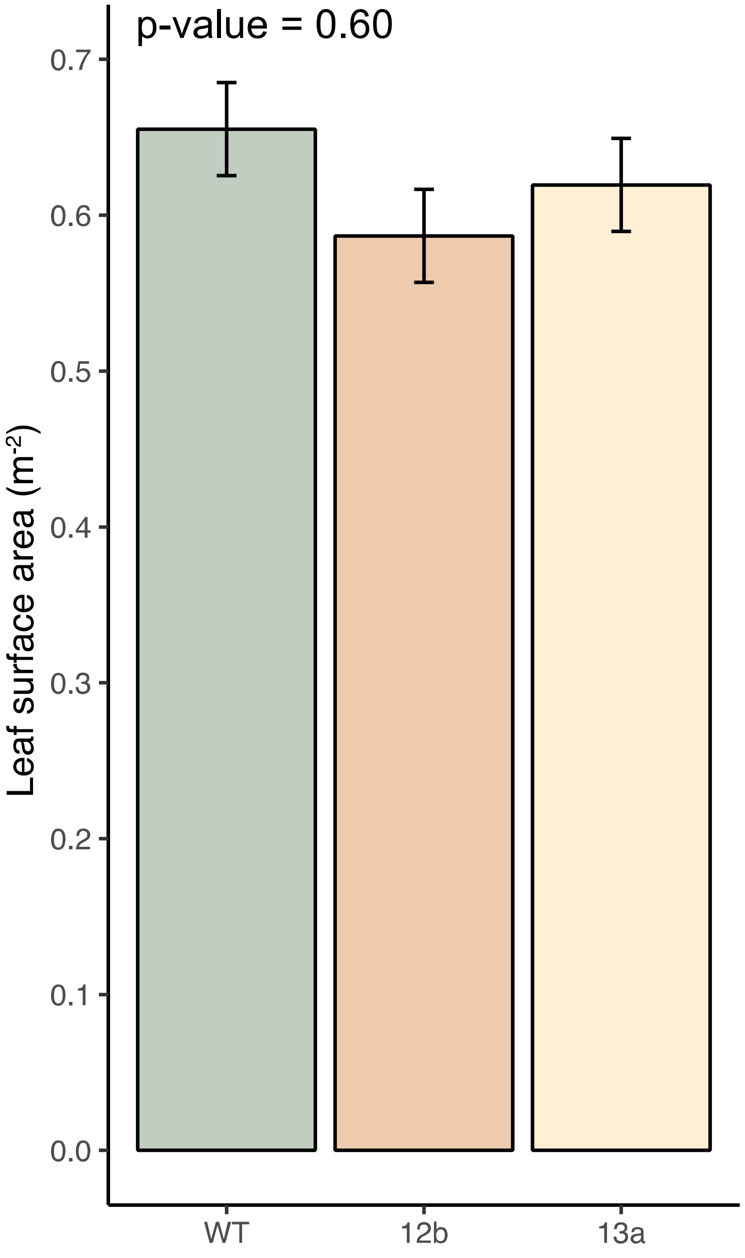

p-value = 0.60
0.7
0.6
0.5
0.4
0.3
0.2
0.1
0.0
Leaf surface area (m-2)
WT
12b
13a

Supp. Fig. S8

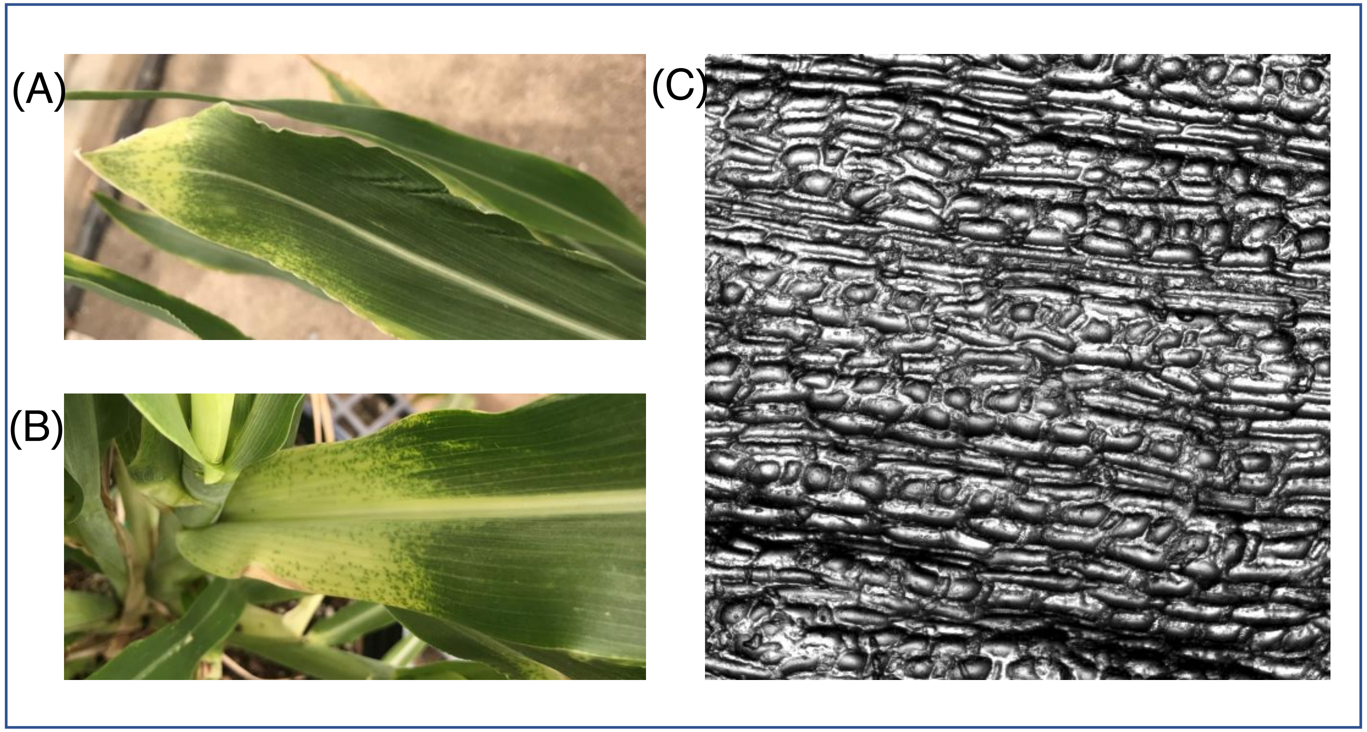

Supplement: erae289_suppl_Supplementary_Table_S1_Figures_S1-S8 [file erae289_suppl_supplementary_table_s1_figures_s1-s8.pdf]
